# Leaf herbivory counteracts nematode-triggered repression of jasmonate-related defenses in tomato roots

**DOI:** 10.1093/plphys/kiab368

**Published:** 2021-08-26

**Authors:** Ainhoa Martínez-Medina, Crispus M Mbaluto, Anne Maedicke, Alexander Weinhold, Fredd Vergara, Nicole M van Dam

**Affiliations:** 1 Molecular Interaction Ecology, German Centre for Integrative Biodiversity Research (iDiv) Halle-Jena-Leipzig, Puschstraße 4, 04103 Leipzig, Germany; 2 Institute of Biodiversity, Friedrich Schiller University Jena, Dornburgerstraße 159, 07743 Jena, Germany; 3 Plant-Microorganism Interactions, Institute of Natural Resources and Agrobiology of Salamanca (IRNASA‐CSIC), Cordel de Merinas 40-52, 37008 Salamanca, Spain

## Abstract

Shoot herbivores may influence the communities of herbivores associated with the roots via inducible defenses. However, the molecular mechanisms and hormonal signaling underpinning the systemic impact of leaf herbivory on root-induced responses against nematodes remain poorly understood. By using tomato (*Solanum lycopersicum*) as a model plant, we explored the impact of leaf herbivory by *Manduca sexta* on the performance of the root knot nematode *Meloidogyne incognita*. By performing glasshouse bioassays, we found that leaf herbivory reduced *M. incognita* performance in the roots. By analyzing the root expression profile of a set of oxylipin-related marker genes and jasmonate root content, we show that leaf herbivory systemically activates the 13-Lipoxigenase (LOX) and 9-LOX branches of the oxylipin pathway in roots and counteracts the *M. incognita*-triggered repression of the 13-LOX branch. By using untargeted metabolomics, we also found that leaf herbivory counteracts the *M. incognita*-mediated repression of putative root chemical defenses. To explore the signaling involved in this shoot-to-root interaction, we performed glasshouse bioassays with grafted plants compromised in jasmonate synthesis or perception, specifically in their shoots. We demonstrated the importance of an intact shoot jasmonate perception, whereas having an intact jasmonate biosynthesis pathway was not essential for this shoot-to-root interaction. Our results highlight the impact of leaf herbivory on the ability of *M. incognita* to manipulate root defenses and point to an important role for the jasmonate signaling pathway in shoot-to-root signaling.

## Teaser

Leaf herbivory counteracts the repression of jasmonate-related defenses triggered by a root knot nematode in tomato roots impairing the nematode performance via shoot-to-root jasmonate signaling

## Introduction

Plants are constantly subject to a range of detrimental organisms that attack above and belowground plant parts. To prevent insect herbivore damage, plants activate their defense arsenal upon recognition of the attacker encountered (Pieterse et al., 2009). Plant anti-herbivore defense responses include, among others, the production of defensive proteins and toxic metabolites that impact the herbivore’s preference, feeding rate and/or development (Erb and Reymond, 2019; Erb and Kliebenstein, 2020). Herbivore-induced defense responses are regulated by a network of interconnected signaling pathways in which plant hormones play a major regulatory role (Erb and Reymond, 2019). Among them, the jasmonates, a family of oxylipins, emerged as key signals in plant responses to insect chewing herbivores, such as beetles and caterpillars (Howe and Jander, 2008). Other hormones, such as salicylic acid, abscisic acid, ethylene and auxins, may interact with the jasmonate signaling pathway in the orchestration of plant defenses against herbivores (Erb et al., 2012; Machado et al., 2016). Herbivore-induced defenses are typically expressed not only locally in the damaged tissue, but also systemically in undamaged plant parts that are spatially separated from the inducer (Heil and Ton, 2008). Such a systemic response enables plants to protect undamaged tissues from herbivory, and can influence the performance of other organisms that are feeding on the same plant, either simultaneously or later in time (Karban and Baldwin 1997; Soler et al., 2007, 2008). Consequently, plants modulate interactions between hervivorous insects that rarely come into direct physical contact with one another (Bezemer and van Dam, 2005; Soler et al., 2013).

The majority of studies on plant-mediated interactions between herbivores are constrained to aboveground tissues. However, a growing body of evidence shows that plant-mediated interactions via changes in inducible defenses also occur between aboveground and belowground organisms (Papadopoulou and van Dam, 2017). Belowground herbivory can increase the level of plant defense compounds, such as terpenoids, glucosinolates, or phenolics in aboveground plant tissues. This can affect herbivorous species feeding aboveground on the same plants (Bezemer et al., 2003, 2004; van Dam et al., 2004, 2005; Hol et al., 2004). Defensive properties of the roots have been less studied compared to aboveground plant parts. However, few studies show that aboveground herbivory can also induce defenses systemically in belowground tissues, affecting plant interaction with root-feeding organisms (Bezemer et al., 2004; Soler et al., 2007; Erb et al., 2015; Machado et al., 2018; Mbaluto et al., 2020, 2021). Several hormones, such as jasmonates, abscisic acid and auxins, play important roles in aboveground–belowground signaling (Erb et al., 2009; Machado et al., 2013; Fragoso et al., 2014; Schulze et al., 2019). However, the mechanisms driving these systemic effects and the long-distance signals involved remain poorly understood. More specifically, very little information is available about the molecular mechanisms and signaling underlying the systemic impact of leaf herbivory on root defensive responses against plant parasites such as root knot nematodes.

Root knot nematodes are parasitic animals able to manipulate plants to produce feeding cells in the roots to supply the nematodes with nutrients (Gheysen and Mitchum, 2011). The infection cycle of root knot nematodes comprises different stages, including the invasion of the host root, followed by the establishment in the root tissues and reproduction. Once the infective second-stage juveniles hatch, they pierce and penetrate the host root near the elongation zone and migrate intercellularly toward the vascular cylinder. There, they establish feeding sites known as giant cells. Hyperplasia and hypertrophy of the surrounding cells lead to the formation of macroscopically visible root knots or galls in which the nematodes are embedded (Kyndt et al., 2014). As obligate endoparasites that complete most of their life cycle within plant roots, the ability of root knot nematodes to maintain their feeding sites relies on continuous modulation of plant defenses (Goverse and Smant, 2014). Several signaling molecules are involved in plant defense responses mounted against root knot nematodes. Among them, jasmonates play a major role in basal and induced defenses against root knot nematodes in a number of plant species (Cooper et al., 2005; Fujimoto et al., 2011; Nahar et al., 2011; Gleason et al., 2016; Hu et al., 2017; Kyndt et al., 2017; Yimer et al., 2018).

Several studies demonstrate that foliar treatment with jasmonic acid (JA) or methyl jasmonate reduces plant susceptibility to root knot nematodes. This indicates that the involvement of jasmonates in the shoot-to-root communication is underlying the systemic protection against root knot nematodes (Cooper et al., 2005; Fujimoto et al., 2011; Nahar et al., 2011; Vieira dos Santos et al., 2013; Fan et al., 2015). However, the specific mechanisms responsible for this phenomenon remain ambiguous. Moreover, studies addressing the impact of aboveground elicitation by shoot herbivory on root knot nematodes infection are scarce and show contrasting results. For instance, transient shoot herbivory by the chewing herbivore *Spodoptera exigua* triggered a decrease in JA levels in tomato (*Solanum lycopersicum*) roots and did not affect the number of galls induced by the root knot nematode *Meloidogyne incognita* (Kafle et al., 2017). By contrast, simulated herbivory by *Manduca sexta* strongly induced jasmonates in the root of *Nicotiana attenuata* plants and led to an increase in the number of *M. incognita* eggs (Machado et al., 2018).

The long-term root interaction with root knot nematodes is highly complex and dynamic. The outcome of the interaction between nematodes and the plant results from the continuous interplay between the active manipulation of host defenses by nematode effectors secreted in the plant tissue to promote susceptibility, and defense responses triggered by the plant to control the infection (Goverse and Bird, 2011; Goverse and Smant, 2014; Ibrahim et al., 2019; Mbaluto et al., 2020). Accordingly, we hypothesized here that the systemic elicitation of root defenses by leaf herbivory counteracts the ability of the root knot nematode *M. incognita* to manipulate root defenses, thereby negatively affecting its infection success. By performing a series of glasshouse bioassays, we found that continuous leaf herbivory by the chewing insect *Ma. sexta* impaired nematode performance. By analyzing the expression profiles of oxylipin-related genes combined with targeted and untargeted metabolomics, we showed that *Ma. sexta* leaf herbivory counteracts the ability of *M. incognita* to downregulate jasmonate-related root defenses. To further explore the signaling involved in the shoot-to-root interaction, we performed bioassays with grafted plants compromised in jasmonate synthesis or signaling in their shoots. We demonstrated the importance of intact shoot jasmonate signaling, whereas *de novo* shoot jasmonate biosynthesis was not required to enhance resistance against *M. incognita*. Our results highlight the impact of leaf herbivory on the ability of *M. incognita* to manipulate root defenses, and point to an important role for jasmonate signaling in shoot-to-root signaling.

## Results

### Shoot herbivory by *Ma*. *sexta* reduces *M*. *incognita* performance

**Figure 1:**
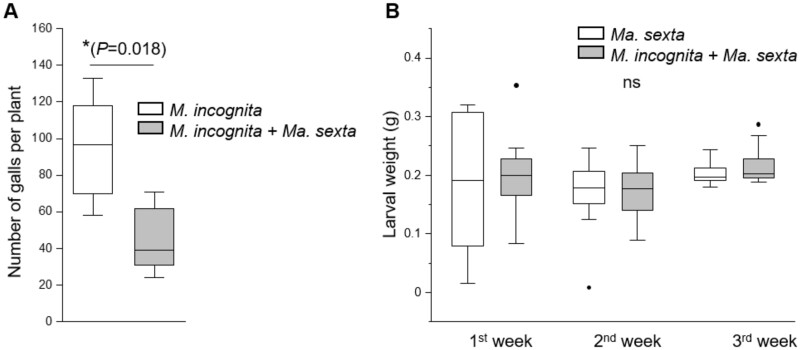
The impact of *Ma. sexta* leaf herbivory on the number of *M. incognita* root galls, and the impact of *M. incognita* root infection on *Ma. sexta* larval weight. Tomato plants were challenged with *M. incognita* eggs or *Ma. sexta* larvae, or with both herbivores at the same time. A, Three weeks later the number of galls was counted in plants challenged with *M. incognita* alone and in roots of plants that were also challenged with *Ma. sexta* feeding on the shoots (*M. incognita + Ma. sexta*). B, During the bioassay (three weeks), *Ma. sexta* larvae were replaced weekly with newly hatched neonates to avoid the consumption of the entire shoot biomass. The weight of *Ma. sexta* larvae was recorded at the end of every weekly feeding period (first week, second week, and third week). The weight of *Ma. sexta* larvae feeding on plants that were challenged with *Ma. sexta* alone (*Ma. sexta*) and larvae feeding on plants that were also root-inoculated with *M. incognita* eggs (*Ma. sexta + M. incognita*) was recorded. Box plots represent the interquartile range (IQR), the bisecting line represents the median, the whiskers represent 1.5 times the IQR, the dots represent outlier points, and the data are from 10 individual plants. In (A), the asterisk indicates significant differences between the treatments according to Student’s *t* test (*P* ≤* *0.05). In (B), ns: not significant.

We first studied whether continuous leaf herbivory by *Ma. sexta* impacts the performance of *M. incognita*. We challenged tomato plants with *M. incognita* alone, or with both *Ma. sexta* and *M. incognita*, and three weeks later we evaluated the number of root galls. Shoot herbivory by *Ma. sexta* led to a reduction (up to 50%) in the number of root galls induced by *M. incognita* (Figure 1A), indicating that *Ma. sexta* leaf herbivory impairs *M. incognita* root infection. We further recorded the weight of *Ma. sexta* larvae after one week of feeding on plants that were inoculated or not inoculated with *M. incognita*. We recorded the weight of *Ma. sexta* larvae at the end of every weekly feeding period of the bioassay. We found that *M. incognita* inoculation did not affect *Ma. sexta* larval weight during the duration of the experiment (Figure 1B, Supplemental Table S1).

### *Manduca sexta* leaf herbivory counteracts the repression of the 13-LOX oxylipin pathway mediated by *M*. *incognita* infection in tomato roots

**Figure 2:**
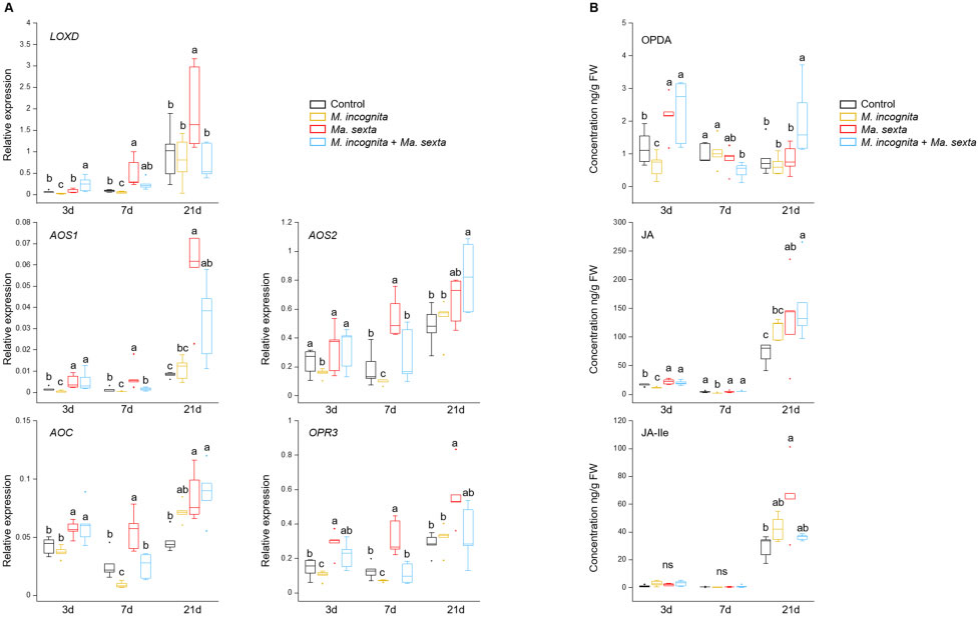
*Ma. sexta* leaf herbivory antagonizes the repression of the 13-LOX oxylipin pathway triggered by *M. incognita* in tomato roots. A, Expression levels of the 13-LOX biosynthesis marker genes *LOXD* (*LIPOXYGENASE D*)*, AOS1* (*ALLENE OXIDE SYNTHASE 1*)*, AOS2* (*ALLENE OXIDE SYNTHASE 2*)*, AOC* (*ALLENE OXIDE CYCLASE*), and *OPR3* (*12-OXOPHYTODIENOIC ACID REDUCTASE 3*) and (B) root levels of OPDA, JA, and JA-Ile. Gene expression and metabolite contents were analyzed in roots of plants that were challenged with *M. incognita* or *Ma. sexta* alone or in combination, and in unchallenged control plants. Gene expression and metabolite contents were analyzed 3, 7, and 21 d after *M. incognita* inoculation. Box plots represent the IQR, the bisecting line represents the median, the whiskers represent 1.5 times the IQR, the dots represent outlier points, and the data are from five individual plants. In (A), the results are normalized to *SlEF* gene expression levels. At each specific time point, different letters indicate differences between treatments (ANOVA, Tukey’s test; *P *≤* *0.05). ns: not significant.

**Figure 3:**
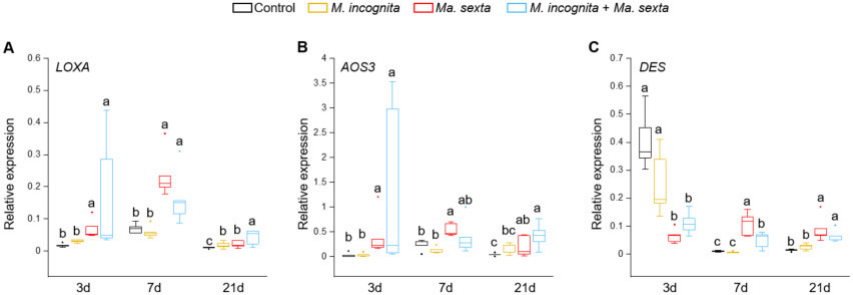
Impact of *M. incognita* and *Ma. sexta* on the 9-LOX oxylipin pathway in tomato roots. Expression levels of the 9-LOX biosynthesis marker genes (A) *LOXA* (*LIPOXYGENASE A*), (B) *AOS3* (*ALLENE OXIDE SYNTHASE 3*), and (C) *DES* (*DIVINYL ETHER SYNTHASE*). Gene expression was analyzed in roots of plants that were challenged with *M. incognita* or *Ma. sexta* alone or in combination, and in unchallenged control plants. Gene expression was analyzed at 3, 7, and 21 d after *M. incognita* inoculation. Box plots represent the IQR, the bisecting line represents the median, the whiskers represent 1.5 times the IQR, the dots represent outlier points, and the data are from five individual plants. Results were normalized to the *SlEF* gene expression levels. At each specific time point, different letters indicate differences between treatments (ANOVA, Tukey’s test *P *≤* *0.05).

Root knot nematodes can modulate oxylipin-related root defenses to successfully parasitize their host (Gheysen and Mitchum, 2019). To understand whether leaf herbivory by *Ma. sexta* interferes with the ability of *M. incognita* to modulate the oxylipin pathway, we first studied the impact of *M. incognita* infection on the oxylipin pathway in tomato roots, during different stages of the nematode infection cycle: invasion (3 d after inoculation), gall-induction (7 d after inoculation) and reproduction (21 d after infection) stages. In tomato, there are two major branches of the oxylipin pathway; the 13-LOX branch and the 9-LOX branch (Itoh et al., 2002; Howe et al., 2018). We found a general transcriptional downregulation of the genes *LOXD* (*LIPOXYGENASE D*)*, AOS1* (*ALLENE OXIDE SYNTHASE 1*)*, AOS2* (*ALLENE OXIDE SYNTHASE 2*)*, AOC* (*ALLENE OXIDE CYCLASE*), and *OPR3* (*12-OXOPHYTODIENOIC ACID REDUCTASE 3*), encoding for key enzymes of the 13-LOX branch, in *M. incognita* infected roots (Figure 2A, Supplemental Table S2). This downregulation was specifically observed at 3 and 7 d after nematode inoculation, coinciding with the invasion and gall-induction stages of the *M. incognita* infection cycle. However, at 21 d after nematode inoculation there were no significant differences in root expression of *LOXD*, *AOS1*, *AOS2*, *AOC*, and *OPR3* between *M. incognita* inoculated and control plants (Figure 2A). Along the same lines, *M. incognita* root inoculation led to a downregulation of the JA-responsive marker gene *PI II* (*PROTEINASE INHIBITOR II*) specifically at 7 d after the inoculation, and *LAPA (LEUCINE AMINOPEPTIDASE A)* at 3 and 7 d after the inoculation (Supplemental Figure S1). Moreover, *M. incognita* root inoculation led to a general reduction of OPDA (oxophytodienoic acid) and JA levels in tomato roots at the early stages of nematode infection (Figure 2B, Supplemental Table S2). The levels of OPDA were reduced in *M. incognita* infected roots 3 d after inoculation, while JA levels were reduced both at 3 and 7 d after inoculation. Root levels of OPDA and JA in *M. incognita*-inoculated roots were similar to those found in control roots 21 d after *M. incognita* inoculation (Figure 2B). *Meloidogyne incognita* inoculation did not significantly affect JA-Ile (jasmonoyl-isoleucine) levels in tomato roots (Figure 2B). In contrast to the 13-LOX branch, *M. incognita* inoculation overall, did not significantly affect the transcription levels of *LOXA* (*LIPOXYGENASE A*)*, AOS3* (*ALLENE OXIDE SYNTHASE 3*), and *DES* (*DIVINYL ETHER SYNTHASE*), encoding key enzymes of the 9-LOX branch (Figure 3). Only in the specific case of *LOXA*, we found that *M. incognita* root infection triggered a higher expression at 21 d (Figure 3A). Our results indicate that *M. incognita* infection led to an early and transient downregulation of the 13-LOX branch of oxylipin pathway in tomato roots.

Next, we studied whether *Ma. sexta* leaf herbivory systemically affects the *M. incognita*-mediated downregulation of the 13-LOX branch of the oxylipin pathway. *Ma. sexta* leaf herbivory systemically triggered a general transcriptional activation of the 13-LOX branch marker genes *LOXD*, *AOS1*, *AOS2*, *AOC*, and *OPR3* in plant roots (Figure 2A, Supplemental Table S2). Shoot herbivory also systemically increased the levels of OPDA, JA and JA-Ile in tomato roots, although this effect was time- and hormone-dependent (Figure 2B, Supplemental Table S2). Root OPDA levels increased 3 d after shoot herbivory, JA root levels increased at 3 and 21 d after shoot herbivory, while JA-Ile levels increased at 21 d (Figure 2B). Remarkably, in roots of plants that were challenged with both *Ma. sexta* and *M. incognita*, transcript levels of *LOXD*, *AOS1*, *AOS2*, *AOC*, and *OPR3*, overall, were higher than those in roots of plants inoculated with *M. incognita* alone (Figure 2A). It is noticeable that an additive effect was observed for *LOXD* expression. At 3 d after inoculation, plants challenged with both herbivores showed higher levels of *LOXD* expression compared to plants challenged with *M. incognita* or *Ma. sexta* alone (Figure 2A). Moreover, at 7 d, we found that the expression of *LOXD*, *AOS1*, *AOS2*, *AOC*, and *OPR3* in plants challenged with both herbivores was in between the expression levels found in plants challenged with *M. incognita* or *Ma. sexta* alone (Figure 2A). In accordance with the gene expression levels, the contents of OPDA and JA in plants challenged with both *Ma. sexta* and *M. incognita* were generally higher than those observed in plants challenged with *M. incognita* alone (Figure 2B). The levels of JA-Ile in plants challenged with *Ma. sexta* and *M. incognita* remained similar to those in control plants.

*Ma. sexta* leaf herbivory generally increased root expression of the 9-LOX branch-marker genes *LOXA*, *AOS3*, and *DES* (Figure 3, Supplemental Table S2). Root expression of *LOXA* and AOS3 was upregulated by *Ma. sexta* compared to controls, over the duration of the experiment (Figure 3, A and B); while *DES* was upregulated by *Ma. sexta* specifically at 7 and 21 d after *Ma. sexta* challenge (Figure 3C). By contrast, *Ma. sexta* herbivory led to a downregulation of *DES* specifically at 3 d after onset of *Ma. sexta* herbivory (Figure 3C). In plants that were challenged with *Ma. sexta* and *M. incognita* together, the expression levels of *LOXA*, *AOS3*, and *DES* were overall similar to the level observed in plants challenged with *Ma. sexta* alone. However, in the case of *LOXA* we found an additive effect at 21 d. Indeed, the expression of *LOXA* in plants challenged with both herbivores at 21 d was higher than in plants challenged with *Ma. sexta* or *M. incognita* alone (Figure 3A). Altogether, our results show that *Ma. sexta* leaf herbivory systemically activates the 13-LOX and 9-LOX branches of the oxylipin pathway in tomato roots, and counteracts the *M. incognita*-triggered repression of the 13-LOX branch.

### Leaf herbivory by *Ma. sexta* alters the root metabolic signature triggered by *M. incognita* infection

**Figure 4:**
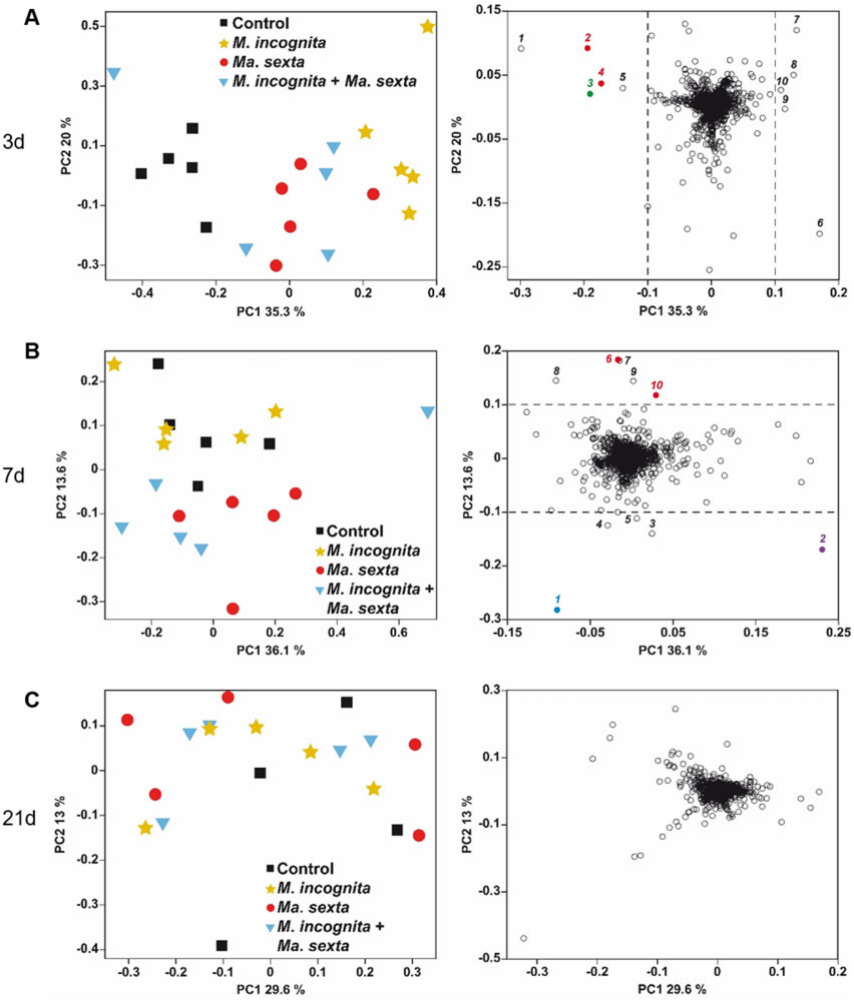
Impact of *M. incognita* and *Ma. sexta* on the metabolic profile of tomato roots. PCA of the tomato root metabolome. Plants were challenged with *M. incognita* or *Ma. sexta* alone or in combination, or not challenged (control). Metabolite profiles were analyzed at (A) 3 d, (B) 7 d, and (C) 21 d after *M. incognita* inoculation. Left: score plots, right: loading plots of features defined by their retention time and *m*/*z* value after mass spectral signal alignment. The dotted horizontal lines indicate the ±0.1 loading thresholds for features separating treatments on PC1 at 3 d (A, right column). Similarly, the dotted horizontal lines in (B), right column, indicate the ±0.1 loading thresholds for features separating treatments on PC2 at 7 d. Features with loadings greater than these thresholds are numbered and listed in Supplemental Table S3. Features belonging to a predicted molecule appear in color: blue: α-tomatine (number 1 in B); purple: α-dehydrotomatine (number 2 in B); red: a phenylpropanoid-polyamine conjugate (numbers 2 and 4 in A; 6 and 10 in B); and green: a chlorogenic acid dimer (number 3 in A). Features without a predicted molecule are shown in black.

The oxylipin pathway is involved in the regulation of a plethora of secondary metabolites in plants (Wasternack and Strnad, 2019). We next investigated whether the systemic impact of *Ma. sexta* leaf herbivory on root oxylipins was associated with changes in the root metabolome. We performed an untargeted metabolomics analysis of roots upon shoot and root herbivory. Principal component analysis (PCA) was used to reveal differences in the metabolic profiles among treatments. At 3 d after nematode inoculation, the metabolomes of roots inoculated with *M. incognita* separated from those of control roots, *Ma. sexta* roots and *M. incognita* plus *Ma. sexta* roots on PC1 (Figure 4A). *Ma. sexta* roots and *M. incognita* plus *Ma. sexta* roots separated from control roots on PC1, but their metabolomes greatly overlapped (Figure 4A). At 7 d after *M. incognita* inoculation, there was no clear separation between roots of plants inoculated with *M. incognita* and control plants (Figure 4B). However, the metabolomes of control and *M. incognita* roots separated from *Ma. sexta* roots and partially from *M. incognita* plus *Ma. sexta* roots on PC2 (Figure 4B). There was not a clear separation between the metabolomes of *Ma. sexta* roots and *M. incognita* plus *Ma. sexta* roots (Figure 4B). At 21 d after nematode inoculation, the root metabolomes of the different treatment groups were not separated in the PCA (Figure 4C). Our data indicate that at early time points (3 and 7 d) *Ma. sexta* leaf herbivory systemically impacts the root metabolome, thereby altering the changes triggered by *M. incognita* infection.

### Leaf herbivory by *Ma*. *sexta* counteracts the repression of putative root chemical defenses triggered by *M*. *incognita* infection

To predict metabolite structures that could explain the differences among the treatments (Figure 4), we interpreted the mass spectra of the r.t.−*m/z* features (hereafter *m/z* features) whose loadings had the highest projections on the PC axis on which the treatment groups separated (PC1 at 3 d; PC2 at 7 d; Figure 4). We set an arbitrary threshold of ±0.1 on the respective axes; all loadings with values > 0.1 and ˂ -0.1 were selected for mass spectra interpretation, giving a total of 10 *m/z* features both at 3 and 7 d (Supplemental Table S3). Based on the mass spectra of these *m/z* features, we assigned putative molecular structures when possible. Thus, we could tentatively assign several *m/z* features to the four following compounds: α-tomatine; α-dehydrotomatine; a phenylpropanoid-polyamine conjugate and a chlorogenic acid dimer (Supplemental Figure S2). In addition, we found seven *m/z* features at 3 d and six *m/z* features at 7 d to differ among the treatments that we were unable to assign to compound structures (Supplemental Table S3, Supplemental Figures S3, S4).

**Figure 5:**
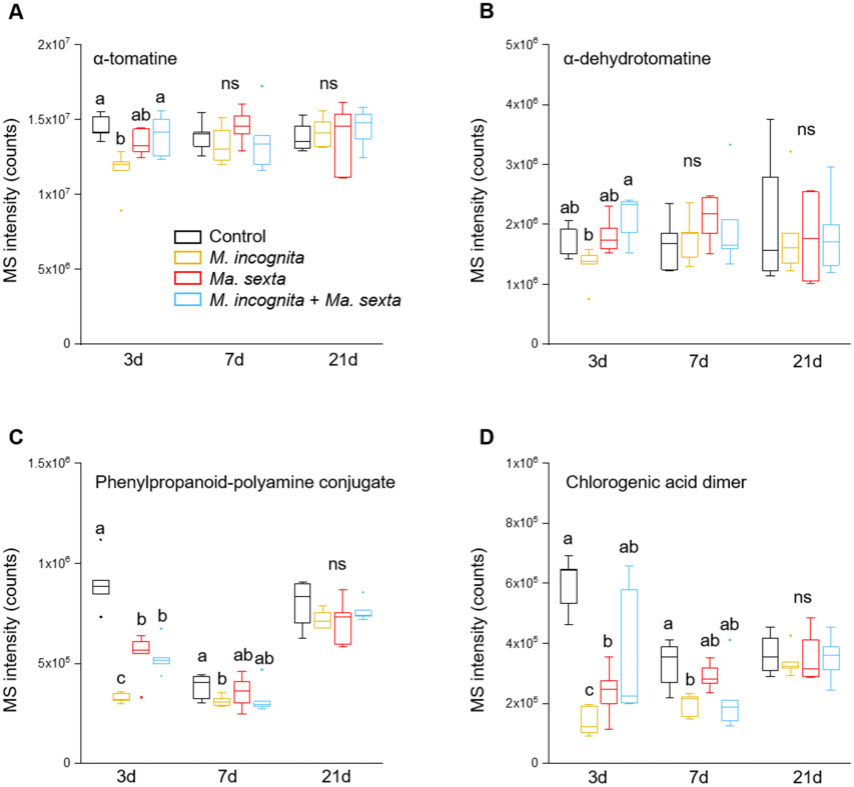
*Ma. sexta* leaf herbivory antagonizes the repression of defense-related metabolites triggered by *M. incognita.* Relative intensity of selected *m*/*z* features, of the metabolites (A) α-tomatine, (B) α-dehydrotomatine, (C) a phenylpropanoid-polyamine conjugate, and (D) a chlorogenic acid dimer. The metabolites were tentatively identified in roots of tomato plants that were challenged with *M. incognita* or *Ma. sexta* alone or in combination, and in unchallenged control plants at 3, 7, and 21 d after *M. incognita* inoculation. Box plots represent the IQR, the bisecting line represents the median, the whiskers represent 1.5 times the IQR, the dots represent outlier points, and the data are from five individual plants. At each specific time point, different letters indicate differences among treatments (ANOVA, Tukey’s test *P *≤* *0.05). ns: not significant.

To study the impact of *M. incognita* and *Ma. sexta* on the relative concentrations of the metabolites, we focused specifically on those *m/z* features for which we were able to predict their structure. First, we studied whether *M. incognita* infection affected the levels of these predicted metabolites in tomato roots. We compared the intensity of one diagnostic fragment for each predicted molecule (α-tomatine *m/z* 578.4056, rt 8.65 min; α-dehydrotomatine *m/z* 576.3901, rt 8.41 min; the phenylpropanoid-polyamine conjugate *m/z* 163.0601, rt 0.98 min; and the chlorogenic acid dimer *m/z* 163.0387 rt 5.25 min). *Meloidogyne incognita* root infection led to a decrease in the level of the steroidal glycoalkaloid α-tomatine, and less so of α-dehydrotomatine, in tomato roots at 3 d after inoculation (Figure 5, A and B; Supplemental Table S2). At 7 and 21 d after the inoculation, α-tomatine and α-dehydrotomatine levels in *M. incognita* infected roots were similar to that in control roots (Figure 5, A and B). *Meloidogyne incognita* infection also led to a decreased level of a phenylpropanoid-polyamine conjugate and a chlorogenic acid dimer at 3 and 7 d, whereas no significant differences were found between *M. incognita* and control roots at 21 d (Figure 5, C and D). These results indicate that *M. incognita* root infection led to a decrease in the levels of α-tomatine, α-dehydrotomatine, a phenylpropanoid-polyamine conjugate, and a chlorogenic acid dimer in tomato roots during the early stages of the infection.

We next studied whether *Ma. sexta* leaf herbivory systemically affects the *M. incognita*-mediated repression of the predicted defense-related metabolites in tomato roots. *Ma. sexta* herbivory alone did not significantly affect the root levels of α-tomatine or α-dehydrotomatine (Figure 5, A and B). Remarkably, *Ma. sexta* leaf herbivory led to a decrease in the levels of the phenylpropanoid-polyamine conjugate (Figure 5C) and the chlorogenic acid dimer (Figure 5D) at 3 d after herbivory, but to a lesser extent than *M. incognita* infection. This systemic effect was gone at 7 and 21 d of *Ma. sexta* feeding (Figure 5, C and D). When plants were challenged with both *Ma. sexta* and *M. incognita*, *M. incognita* infection failed partially or completely in reducing the levels of the analyzed metabolites (Figure 5). Moreover, the level of the analyzed root metabolites in plants challenged with both herbivores was more similar to those observed in roots of plant challenged with *Ma. sexta* alone, than to those in plants challenged with *M. incognita* alone (Figure 5).

### Shoot jasmonates perception but not *de novo* synthesis is required for *Ma*. *sexta* systemic impairment of *M. incognita* performance

**Figure 6:**
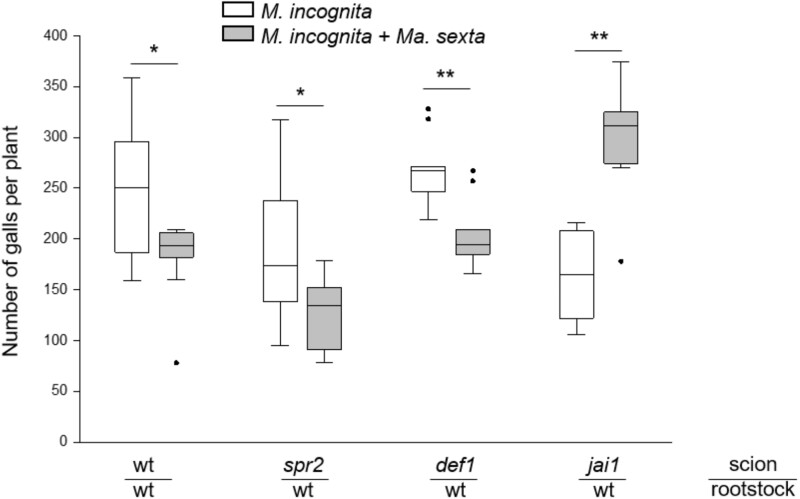
The involvement of *de novo* shoot jasmonate synthesis and jasmonate perception in shoot-to-root *Ma. sexta-M. incognita* interaction. Grafts were made with rootstocks of the wt Castlemart and scions of the wt Castlemart (wt/wt), the jasmonate biosynthesis compromised lines *spr2 (spr2*/wt*)* and *def1* (*def1*/wt) or the jasmonate perception compromised line *jai1 (jai*/wt). One week after grafting, the plants were root inoculated with *M. incognita*. Half of the plants were also challenged aboveground with *Ma. sexta.* Three weeks after *M. incognita* inoculation the number of galls was evaluated. X-axis shows the graft combinations (scion/rootstock). Box plots represent the IQR, the bisecting line represents the median, the whiskers represent 1.5 times the IQR, the dots represent outlier points, and the data are from 8 to 10 individual plants. For each graph type, the asterisks indicate significant differences between treatments according to Student’s t test (*P ≤0.05; **P ≤ 0.01).

We next studied whether the impact of *Ma. sexta* feeding on *M. incognita* performance is mediated by jasmonate shoot-to-root signaling. We used grafted plants with compromised jasmonate synthesis or perception, specifically in their shoots. We first observed that in absence of Ma. sexta, the number of root galls in grafts composed by wild-type (wt) rootstock and scions of the mutant *spr2* (*suppressor of prosystemin‐mediated responses2*; compromised in wound-induced JA biosynthesis), or *jasmonic acid-insensitive1 [jai1]*; compromised in jasmonate-perception) was reduced, compared to the numbers of galls observed in wt/wt grafted plants (Figure 6; *P *= 0.048 and *P *= 0.007, respectively). On the other hand, a similar number of root galls was observed in wt/wt grafted plants and in grafts composed by wt rootstock and scions of *defenseless-1 [def1]*; defective in the octadecanoid synthesis pathway). These results point to a role of shoot JA biosynthesis and signaling in root susceptibility to *M. incognita* in this plant–nematode interaction. We further observed that in in wt/wt grafted plants *Ma. sexta* herbivory reduced the number of *M. incognita* root galls as occurred in non-grafted plants (Figures 1A and 6). Similarly, a reduction in the number of root galls upon leaf feeding by *Ma. sexta* was observed in grafts composed by wt rootstock and scions of the mutant *spr2* or *def1* (Figure 6). In contrast, on *jai1*/wt grafts *Ma. sexta* treatment failed in reducing the number of *M. incognita* root galls. In fact, the number of root galls increased on *Ma. sexta*-challenged *jai1*/wt grafts compared to non-*Ma. sexta* treatment (Figure 6). These observations indicate that *de novo* jasmonate biosynthesis in shoots is not required for the *Ma. sexta-*triggered impairment of *M. incognita* performance. However, an intact jasmonate perception seems to be essential.

### Shoot herbivory by *Ma*. *sexta* delays *M. incognita* root invasion and impedes its development and fecundity

**Figure 7:**
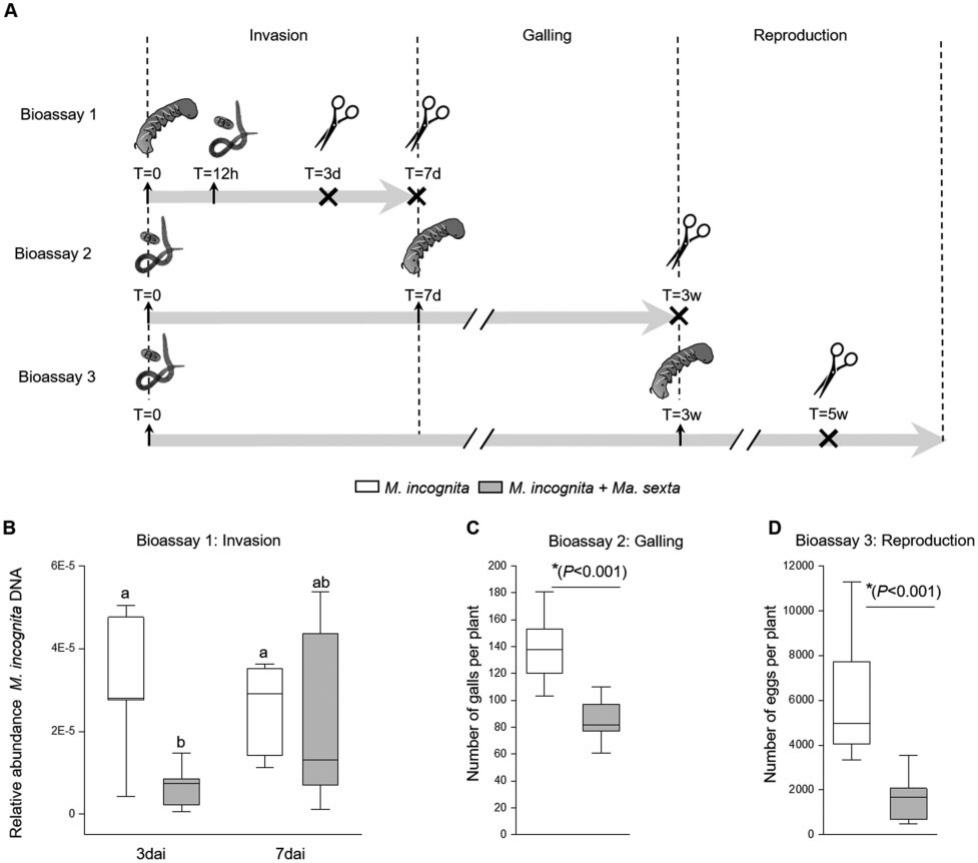
*Ma. sexta* leaf herbivory affects the *M. incognita* infection cycle. A, Schematic overview of the experimental designs used to evaluate the impact of *Ma. sexta* leaf herbivory on specific stages of the *M. incognita* infection cycle. B, The impact of *Ma. sexta* leaf herbivory on *M. incognita* root invasion was assessed in Bioassay 1. Infection success of *M. incognita* was quantified at 3 and 7 d after *M. incognita* inoculation (dai) by analyzing *M. incognita Actin* gene relative to *SlEF* gene. This was done in roots of plants challenged with *M. incognita* alone (*M. incognita)* and in roots of plants that were also challenged with *Ma. sexta* (*M. incognita + Ma. sexta*). C, The impact of *Ma. sexta* leaf herbivory on *M. incognita* galling was assessed in Bioassay 2. The number of galls was quantified in roots of plants challenged with *M. incognita* alone and in roots of plants that were also challenged with *Ma. sexta*, three weeks after *M. incognita* inoculation. D, The impact of *Ma. sexta* leaf herbivory on *M. incognita* reproduction was analyzed in Bioassay 3. The total number of number of eggs was assessed in the roots of plants challenged with *M. incognita* alone and in roots of plants that were also challenged with *Ma. sexta*. Eggs were collected from tomato root tissue five weeks after *M. incognita* inoculation. Box plots represent the IQR, the bisecting line represents the median, the whiskers represent 1.5 times the IQR, and the data are from five individual plants in B, or 10 in C and D. In (B), Different letters indicate differences between treatments (ANOVA, Tukey’s test *P *≤* *0.05). In (C) and (D), the asterisk indicates significant differences between the treatments according to Student’s *t* test (*P *≤* *0.05).

We found that *Ma. sexta* leaf herbivory reduced *M. incognita* performance (Figure 1A). The infection cycle of *M. incognita* comprises root invasion, formation of the feeding sites (galling), and the reproduction stage (Goverse and Smant, 2014). We thus aimed to explore which of the different stages of the nematode infection life cycle are indeed affected by leaf herbivory. With this aim, we performed three different glasshouse bioassays in which we manipulated the specific timing of the shoot and root challenge (Figure 7A). In the first bioassay, we assessed the impact of *Ma. sexta* leaf herbivory on *M. incognita* root invasion (Figure 7A, bioassay 1). At 3 d after nematode inoculation, we found a decrease in *M. incognita* DNA in *M. incognita*-infected roots of plants that were also challenged with *Ma. sexta* when compared to roots of plants challenged with *M. incognita* alone (Figure 7B;Supplemental Table S1). However, at 7 d after nematode inoculation, *M. incognita* DNA levels were similar in roots of plants inoculated with *M. incognita* alone and plants challenged by both *M. incognita* and *Ma. sexta* (Figure 7B). These results indicate that leaf herbivory by *Ma. sexta* delays *M. incognita* root invasion. We next investigated the impact of leaf herbivory on nematode galling (Figure 7A, bioassay 2). *Ma. sexta* leaf herbivory reduced the number of *M. incognita* root galls per root system (Figure 7C), indicating that leaf herbivory impairs the development of the nematodes inside the root tissues. We finally studied whether *Ma. sexta* leaf herbivory affects *M. incognita* fecundity (Figure 7A, bioassay 3). The total number of eggs per plant decreased in the roots of plants challenged with both *M. incognita* and *Ma. sexta*, compared to plants inoculated with *M. incognita* alone (Figure 7D). Collectively, these results show that *Ma. sexta* leaf herbivory reduces *M. incognita* performance by delaying nematode root invasion and by impeding the galling and the reproduction of nematodes inside the roots.

## Discussion

We demonstrated that continuous leaf herbivory by *Ma. sexta* reduces the performance of the root knot nematode *M. incognita* via shoot-to-root interaction. By using a series of manipulative bioassays in which we incorporated the shoot herbivore at different stages of the nematode infection cycle, we found that *Ma. sexta* leaf herbivory delayed *M. incognita* root invasion, and impaired the nematode’s development and fecundity. Several studies have demonstrated that leaf herbivory or shoot elicitation with jasmonates can affect the susceptibility of roots to root knot nematodes. Shoot-induced responses can facilitate or impede nematode performance depending on the study system and the specific performance-related parameters assessed. For instance, *Nicotiana tabacum* shoot defoliation by *Ma. sexta* increased the number of *M. incognita* eggs per gram of root (Kaplan et al., 2008a). Likewise, shoot elicitation with simulated herbivory increased the number of *M. incognita* eggs, while it did not affect the number of galls in *Nicotiana attenuata* plants (Machado et al., 2018). In contrast, a reduction in the number of *M. incognita* galls was found in roots of plants that were previously challenged with aphids (Kafle et al., 2017) or whose shoots were elicited with methyl jasmonate (Nahar et al., 2011; Vieira dos Santos et al., 2013). This shows that interactions between shoot-induced responses and root knot nematodes are highly complex and dynamic (Goverse and Bird, 2011; Goverse and Smant, 2014; Ibrahim et al., 2019). Therefore, such variable results could be attributed to the differences in the study systems, experimental designs and/or sampling times (Wondafrash et al., 2013; van Dam et al., 2018). Here we demonstrated that *Ma. sexta* shoot herbivory negatively affected *M. incognita* throughout the entire nematode infection cycle.

A growing body of evidence demonstrates a pivotal role of jasmonate-regulated defenses in immune responses against root knot nematodes (Cooper et al., 2005; Fujimoto et al., 2011; Nahar et al., 2011; Vieira dos Santos et al., 2013; Fan et al., 2015; Zhao et al., 2015; Zhou et al., 2015; Gleason et al., 2016; Kyndt et al., 2017). Accordingly, we found that *M. incognita* infection repressed the 13-LOX branch of the oxylipin pathway, which leads to the production of jasmonates. This repression was stronger during the early stages of the nematode infection cycle (invasion and induction stages). Previous studies evidenced the ability of root knot nematodes to repress jasmonate-related root defenses at the very early stages after penetration, probably to promote infection success (Barcala et al., 2010; Nahar et al., 2011; Kyndt et al., 2012; Ji et al., 2013; Iberkleid et al., 2015; Gheysen and Mitchum, 2019). Indeed, a stronger repression of *LOXD* was found in tomato roots during the early stages of *Meloidogyne javanica* infection, compared to later stages of the infection (Iberkleid et al., 2015). In contrast to *M. incognita*, *Ma. sexta* leaf herbivory strongly activated jasmonate biosynthesis in roots. It was previously demonstrated that leaf herbivory or mechanical wounding triggers jasmonate-related responses systemically in root tissues (Acosta et al., 2013; Machado et al., 2013, 2018; Larrieu et al., 2015; Schulze et al., 2019). Interestingly, in roots of plants that were co-infected, leaf herbivory prevented the root repression of the jasmonate biosynthesis pathway triggered by *M. incognita* infection. Along the same lines, Nahar et al. (2011) found that shoot elicitation with methyl jasmonate antagonized the *Meloidogyne gaminicola*-induced defense gene repression in roots of rice plants. This means that the shoot-herbivore induced boost of jasmonate-related responses in roots might interfere with the nematode’s ability to manipulate jasmonate-related defenses, leading to a higher plant resistance to nematodes.

Besides jasmonates, the 9-LOX branch of the oxylipin pathway has been associated with plant resistance to root knot nematodes (Gao et al., 2007; Iberkleid et al., 2015). We found that *M. incognita* infection overall, did not significantly affect the expression of the gene markers for the 9-LOX pathway in roots. In contrast, leaf herbivory triggered a general activation of the 9-LOX branch of the oxylipin pathway in roots. Similarly, the roots of plants that were co-infected with both root and leaf herbivores showed an increased activation of the 9-LOX pathway. Several studies support a role of oxylipins produced by the 9-LOX pathway in root defenses. For instance, the 9-LOX derivative 9-hydroxyoctadecatrienoic acid is involved in cell wall modification and ROS signaling in roots (Vellosillo et al., 2007; Marcos et al., 2015). It is therefore conceivable that the shoot-herbivore activation of the 9-LOX pathway in roots could participate in the increased resistance to *M. incognita*. However, the specific role of the 9-LOX branch of the oxylipins pathway in root–nematode interactions is so far unknown.

Jasmonates regulate nearly all biosynthetic pathways leading to defensive metabolites (Wasternack and Strnad, 2019). According to the significant impact of *M. incognita* on root jasmonates, *M. incognita* infection triggered significant changes in root secondary metabolism. Similarly, previous studies revealed the strong impact of parasitic nematodes on the global metabolome of their host plants, including changes in defensive compounds and primary metabolism (Hofmann et al., 2010; Eloh et al., 2016; Machado et al., 2018; Willett et al., 2020). In line with the jasmonate dynamics observed in nematode-infested roots, the impact of *M. incognita* on the root metabolome was stronger during the early stages of infection. Indeed, at 3 d after *M. incognita* inoculation we found root levels of the steroidal glycoalkaloids α-tomatine and α-dehydrotomatine to be reduced. Steroidal glycoalkaloids are jasmonate-regulated defensive compounds with antiherbivore properties (Altesor et al., 2014; Chowański et al., 2016; Abdelkareem et al., 2017; Montero-Vargas et al., 2018; Calf et al., 2020). Though the involvement of steroidal glycoalkaloids on plant–nematode interactions remains ambiguous, several reports reveal the nematicidal activity of other types of alkaloids in different plant species (Thoden et al., 2009; Wang et al., 2012; Jang et al., 2015). These studies suggest that the accumulation of glycoalkaloids may have an important role in root immunity against nematode attack. *Meldoidogyne incognita* infection also decreased the levels of a phenylpropanoid–polyamine conjugate and a chlorogenic acid dimer. In this case, the reduction was also found at later stages of the nematode infection cycle. Polyamines and polyphenols, such as chlorogenic acid, are prominent defense metabolites against a broad range of insect herbivores (Bassard et al., 2010; Kaur et al., 2010; Macoy et al., 2015; Kundu and Vadassery, 2019). Several reports indicate that polyamines and polyphenols are involved in plant resistance against parasitic nematodes (Pegard et al., 2005; Heinick et al., 2010; Hewezi et al., 2010). Although further research would be required to establish whether these responses are indeed adaptive, we hypothesize that the repression of these root metabolites triggered by *M. incognita* infection would favor the nematode’s infection success.

Interestingly, we found that *Ma. sexta* leaf herbivory systemically altered the metabolic root signature triggered by *M. incognita* infection. The impact of shoot herbivory on the global root metabolome has been previously demonstrated (Marti et al., 2013; Gulati et al., 2014; Machado et al., 2018; Mbaluto et al., 2020). Our results further demonstrate the strong influence of aboveground elicitation in the root responses deployed against root herbivores. Indeed, *Ma. sexta* leaf herbivory prevented, totally, or partially, the repression of the accumulation of the defense-related metabolites triggered by early *M. incognita* infection. Along the same lines, several studies have evidenced that herbivory in one plant compartment can suppress the capacity of another herbivore to elicit particular plant responses in a different plant compartment. For instance, Kaplan et al. (2008b) found that leaf herbivory by *Ma. sexta* or *Trichoplusia ni* counteracted the repression in the accumulation of chlorogenic acid triggered in *Nicotiana tabacum* roots by *M. incognita*. Along the same lines, Huang et al. (2017) found that root feeding by *Diabrotica virgifera virgifera* suppressed *Spodoptera frugiperda*-induced root volatile repellents, which led to the maintenance of host attractiveness to *D. v. virgifera*. Although we cannot exclude the possible contribution of alterations in root growth and primary metabolism triggered by the shoot herbivore (Machado et al., 2013), our findings indicate that leaf herbivory can interfere systemically with the ability of root knot nematodes to repress the accumulation of defensive compounds in roots. This may contribute to a stronger anti-nematode defense response in roots of plants infested aboveground with leaf feeding herbivores.

We finally aimed to explore the signaling mechanisms involved in the systemic effect of *Ma. sexta* herbivory on *M. incognita* performance. Jasmonates are important regulatory signals in plant-mediated interactions between leaf- and root-feeding herbivores (Erb et al., 2009; van Dam et al., 2011; Machado et al., 2013; Fragoso et al., 2014; Li et al., 2017; Wang et al., 2019). To assess the involvement of jasmonates in *Ma. sexta*–*M. incognita* shoot-to-root interactions, we used grafted plants compromised in jasmonate biosynthesis or perception. By restraining jasmonate impairment to the shoot, we were able to identify the specific contribution of the aboveground jasmonate pathway in *Ma. sexta*–*M. incognita* shoot-to-root interactions. The grafting experiments showed that the negative impact of *Ma. sexta* herbivory on *M. incognita* infection does not require *de novo* jasmonate biosynthesis in tomato shoots. Leaf herbivory still reduced the number of galls in wt roots grafted with shoots that were compromised in wound-induced jasmonate biosynthesis (*spr2* and *def1*). It is noteworthy that Machado et al. (2018) found that the increased *M. incognita* egg number triggered by simulated shoot herbivory on *Nicotiana attenuata* plants was abolished in *irAOC* plants, which are compromised in jasmonate biosynthesis. However, in their study the authors did not restrain jasmonate biosynthesis impairment to the shoot organs. Therefore, it was not possible to discern whether the shoot-to-root interaction required an intact jasmonate biosynthesis in shoots and/or in roots. In contrast, we found that leaf herbivory did not reduce the number of root galls in grafts with shoots compromised in jasmonate perception (*jai1*). This indicates that for leaf herbivory-triggered root impairment of *M. incognita* infection an intact jasmonate perception is needed in the shoots. The shoot-to-root signaling activity still observed in the *spr2* and *def1* lines could indicate that the residual level of JA or other oxylipins that may accumulate in the shoots of these lines can be sufficient for the shoot-to-root signaling (Li et al., 2003; Zhang et al., 2011; Goetz et al., 2012). Alternatively, it might reflect a JA-independent pathway in the initiation of the shoot-to-root signal. Indeed, several other wound‐induced rapid systemic responses have been described in plants, and may involve oligosaccharides, reactive oxygen species, green leaf volatiles, hydraulic signals, electrical signals, and other plant hormones (Malone, 1993; Leon et al., 2001; Wasternack et al., 2006; Heil and Ton, 2008; Zimmermann et al., 2009; Wang et al., 2019). It is noteworthy that leaf herbivory increased the number of root galls in grafts with shoots compromised in jasmonate perception. Interestingly, leaf herbivory failed in increasing JA and JA-Ile levels in the roots of grafts composed by *jai* scions (Supplemental Figure S5). Moreover, shoot herbivory decreased the level of JA-Ile in the roots of grafts composed by *jai* scions. These findings might further indicate the importance of the systemic induction of jasmonate-regulated root defenses by the shoot herbivore for the impairment of nematode infection. Still, the levels of JA and JA-Ile in the roots of plants that were challenged with the shoot herbivore were similar in both the grafts composed by wt scions and grafts composed by *jai* scions. This suggests that other mechanisms, independent of jasmonate signaling, are also involved in this shoot-to-root interaction. The impact of leaf herbivores on root parasitic nematodes likely depends on the balance between positive effects resulting from increased carbohydrate allocation to the roots (Kaplan et al., 2009; Biere and Goverse 2016) and negative effects resulting from the elicitation of root defenses (Bhattarai et al., 2008; Nahar et al., 2011; Kyndt et al. 2017). Together, our results suggest that an intact jasmonate perception pathway, but not an intact jasmonate biosynthesis pathway, in shoots is required for the negative systemic effect of *Ma. sexta* herbivory on *M. incognita* performance.

## Conclusions

**Figure 8:**
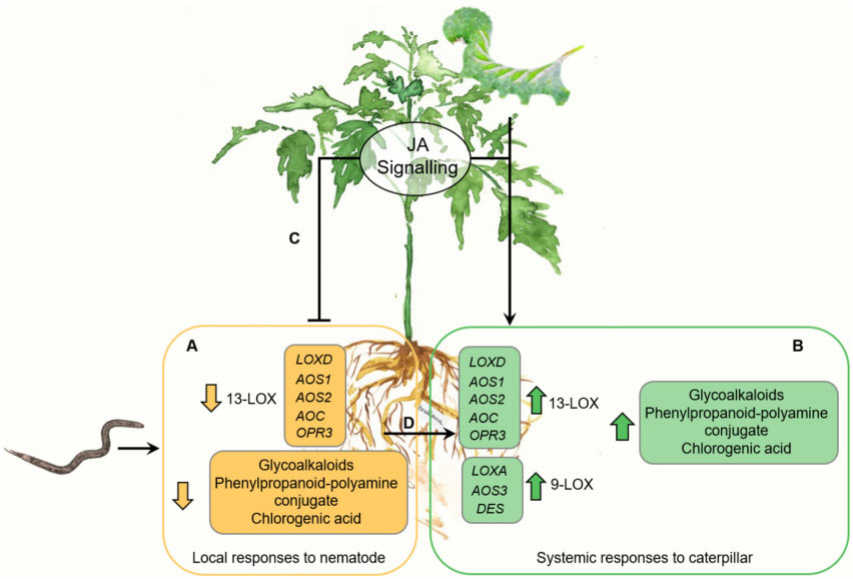
Schematic representation of systemic responses induced by *Ma. sexta* herbivory and their impact on *M. incognita* root-induced responses. A, Root infection by *M. incognita* leads to an early and transient local downregulation of the 13-LOX branch of the oxylipin pathway and the repression of defense-related metabolites in tomato roots. The yellow boxes show specific gene markers for the 13-LOX pathway and defense metabolites that are repressed in the roots upon *M. incognita* infection. B, *Ma. sexta* leaf herbivory triggers a systemic activation of the 13-LOX and 9-LOX branches of the oxylipin pathway, and the accumulation of defense-related metabolites in tomato roots. The green boxes show specific genes markers for the 13-LOX and 9-LOX branches of the oxylipin pathway, and defense metabolites that are enhanced by *Ma. sexta* leaf herbivory in the roots. C, *Ma. sexta* leaf herbivory antagonizes the *M. incognita*-triggered repression of the 13-LOX branch of oxylipin pathway and defense-related metabolites in tomato roots, and leads to a higher nematode resistance. When plants are co-infected with *M. incognita* and *Ma. sexta*, the root responses to *M. incognita* shift from the yellow box to the green box (D). The shoot jasmonate signaling pathway mediates the negative effect of *Ma. sexta* leaf herbivory on *M. incognita* performance. *LOXD*: *LIPOXYGENASE D*; *AOS1*: *ALLENE OXIDE SYNTHASE 1*; *AOS2*: *ALLENE OXIDE SYNTHASE 2*; *AOC*: *ALLENE OXIDE CYCLASE*; and *OPR3*; *12-OXOPHYTODIENOIC ACID REDUCTASE 3*; *LOXA*: *LIPOXYGENASE A*; *AOS3*: *ALLENE OXIDE SYNTHASE*; *DES*: *DIVINYL ETHER SYNTHASE*.

Our study shows that leaf herbivory profoundly alters the defense-related responses triggered in roots by root knot nematodes (Figure 8). Our findings indicate that *Ma. sexta* leaf herbivory interferes, directly or indirectly, with *M. incognita*’s ability to suppress root defenses. In addition, our results highlight the importance of the shoot jasmonate perception pathway and the independence of *de novo* shoot jasmonate biosynthesis in the *Ma. sexta*–*M. incognita* shoot-to-root interaction.

## Materials and methods

### Plant, nematode, and insect material

We used the tomato (*S. lycopersicum*) cultivar Moneymaker unless indicated otherwise. In addition, we used the wild‐type cultivar Castlemart, and its mutant lines *spr2* (*suppressor of prosystemin‐mediated responses2*; Li et al., 2003), the jasmonate-deficient mutant *def1* (Howe et al., 1996) as well as the mutant line *jai1* (Li et al., 2004) compromised in jasmonate signaling. The JA-impaired lines used have been previously reported to be compromised on plant resistance against *Ma. sexta* (Howe et al., 1996; Li et al., 2003; Bosch et al., 2014). Seeds were kindly provided by Prof. Pozo (EEZ-CSIC). We germinated the seeds from Moneymaker and Castlemart, and the lines *spr2* and *def1* for 10 d according to Martínez-Medina et al. (2017). The seeds from the line *jai1* were germinated on a water-saturated filter paper according to Li et al. (2004). We selected homozygous *jai1-1* seedlings from F2 populations according to Li et al. (2004). The inoculum of the root knot nematode *M. incognita* was produced according to Martínez-Medina et al. (2017). We counted and adjusted *M. incognita* eggs to a suspension of 3,000 eggs mL^−l^ water (Martínez-Medina et al., 2017). *Ma. sexta* (Lepidoptera, Sphingidae) eggs were obtained from the Max Planck Institute for Chemical Ecology (Jena, Germany). The *Ma. sexta* culture was maintained according to Grosse‐Wilde et al. (2011).

### Plant growth and experimental design

We transplanted 10-d-old tomato seedlings in 400-mL pots filled with a sterile soil–sand mixture (12:5 v:v) according to Martínez-Medina et al. (2017). We placed the plants in a glasshouse compartment under conditions of 25°C ± 3°C, 16-h light:8-h dark, and 70% relative humidity. Plants were watered three times a week, alternately with tap water and half-strength Hoagland solution (Hoagland and Arnon, 1938). After three weeks, we used the plants for the experiments. For *M. incognita* treatments, we inoculated the plants with approximately 3,000 fresh eggs of *M. incognita* per root by injecting 1 ml of an egg suspension (3,000 eggs mL^−l^) into the soil (Martínez-Medina et al., 2017). Plants that were not assigned to nematode inoculation were mock-inoculated with 1 mL water. For *Ma. sexta* treatments, three neonates were placed on the third fully expanded leaf (counted from below), and allowed to feed freely on the entire plant. We replaced *Ma. sexta* larvae weekly with new neonates to avoid the consumption of the entire shoot biomass. The weight of *Ma. sexta* larvae was recorded at the end of every weekly feeding period. To assess the impact of *Ma. sexta* leaf herbivory on *M. incognita* root infection, plants were inoculated with *M. incognita* eggs and challenged at the same time with the *Ma. sexta* neonates. The bioassay consisted of four treatments: (1) control plants not challenged with any of the herbivores; (2) plants root-inoculated with *M. incognita* eggs; (3) plants exposed to shoot-feeding by *Ma. sexta*; and (4) plants root-inoculated with *M. incognita* eggs and exposed to *Ma. sexta* at the shoot. Ten biological replicates (plants) of each treatment per time-point were used, unless indicated otherwise. At 3, 7, and 21 d after the start of the experiment, the caterpillars were removed and the plants were immediately harvested. Root material of five random replicates was collected and stored at −80°C for molecular and metabolomics analyses. At 21 d after *M. incognita* inoculation, nematode performance was analyzed by counting the total number of galls on root systems.

### Tomato grafts

Seeds from the wt Castlemart and from the jasmonate-compromised lines *spr2*, *def1*, and *jai1* were germinated and grown as described above. Three weeks after transplanting, we grafted scions of the wt Castlemart and from the lines *spr2*, *def1*, and *jai1* onto rootstocks of the wt Castlemart. Grafts were made by cutting the scion and rootstock plants diagonally (∼2 mm above the cotyledon) and securing the junction with a silicone clamp. Grafted plants were placed under 9-h light, 21°C: 15-h dark, 18°C, 90% relative humidity conditions. One week after grafting, the plants were used in the bioassays. All the grafted plants used in the bioassays looked similarly, with not evident differences between them.

### Assessment of nematode behavior

Root systems were carefully washed with tap water. To assess the impact of *Ma. sexta* shoot herbivory on *M. incognita* root invasion, we estimated *M. incognita* biomass by quantitative real-time polymerase chain reaction (qPCR) and the primers of the *Actin* gene from *M. incognita* (Martínez-Medina et al., 2017). Nematode performance was analyzed by counting the number of root galls per plant. Fecundity was determined by counting the total number of eggs according to Martínez-Medina et al. (2017).

### qPCR and RT-qPCR

Total DNA of roots was extracted by using the DNeasy plant kit (Qiagen) according to the manufacturer’s instructions. Total RNA of roots was isolated as described by Oñate‐Sánchez and Vicente‐Carbajosa (2008). We synthesized first-strand cDNA from 1 µg DNase-free RNA using Revert Aid H-minus RT (Thermo Scientific) following the manufacturer’s instructions. We performed qPCR and RT-qPCR reactions according to Papadopoulou et al. (2018), and by using the gene-specific primers described in Supplemental Table S4. For gene expression analysis, the data were normalized by using the housekeeping gene *SlEF* (X14449), encoding the tomato translation elongation factor-1α (Miranda et al., 2013; Martínez-Medina et al., 2017). *Meloidogyne incognita* DNA was estimated by analyzing *M. incognita Actin* gen (MINC06773a) relative to *SlEF* gen.

### Phytohormone extraction and analysis

We extracted root phytohormones from 100 mg of homogenous fresh root material according to Escobar-Bravo et al. (2019), using ethyl acetate containing the internal standards (40 ng D_6_-SA, 40 ng D_6_-ABA, 40 ng D_5_-IAA, 40 ng D_6_-JA, and 40 ng D_6_-JA-Ile) as the solvent. Data acquisition and processing were performed according to Escobar-Bravo et al. (2019). Phytohormone levels were calculated over the amount of fresh mass of plant material (ng^−1^ mg^−1^ fresh weight) and the peak values of the corresponding internal standards.

### Metabolites extraction and data processing

We extracted 100 mg fresh root tissue of each sample as described in Supplemental Methods S1. We performed chromatographic separation of all diluted extracts as described in Supplemental Methods S1. A commercial standard of α-tomatine (Extrasynthese, Lyon, France) was injected using the same conditions but the scan range was modified to 50–1,500 *m*/*z*. Processing of the liquid chromatography mass spectrometry data was performed as described in Supplemental Methods S2. When possible, we produced hypothetical structures based on characteristics like mass fragmentation, presence of inorganic adducts, and comparisons with previously reported mass spectra in MassBank of North America. We normalized the aligned peak areas against the total ion chromatogram. We used the ion intensity values of characteristics signals for each of our predicted structures for comparison of compound abundance in different treatments. The metabolomic data were deposited in the MetaboLights database (Haug et al., 2020).

### Bioassays to evaluate the impact of *Ma. sexta* leaf herbivory on specific stages of the *M. incognita* infection life cycle

To assess the impact of *Ma. sexta* leaf herbivory on each specific stage of *M. incognita* infection, we performed three additional bioassays in the glasshouse, in which we varied the specific timing of shoot and root challenge (Figure 7A). For assessing the impact of *Ma. sexta* leaf herbivory on *M. incognita* root invasion, we placed the *Ma. sexta* larvae on the shoot of the plants, and 12 h later we inoculated the roots with *M. incognita* (Figure 7A, bioassay 1). At 3 and 7 d after *M. incognita* inoculation roots were harvested and stored at −80°C for the quantification of *M. incognita* DNA. For assessing the impact of *Ma. sexta* leaf herbivory on *M. incognita* galling, we first inoculated the plants with *M. incognita*, and 1 week later, after *M. incognita* had successfully invaded the roots, we challenged the plants with *Ma. sexta* larvae (Figure 7A, bioassay 2). Two weeks after challenging the plants with *Ma. sexta* (three weeks after challenging the plants with *M. incognita*), we harvested the plants and visually assessed the number of roots galls. To study the impact of *Ma. sexta* leaf herbivory on *M. incognita* fecundity, we first inoculated the plants with *M. incognita*, and three weeks later (when *M. incognita* had successfully invaded the roots and developed inside), we challenged the plants with *Ma. sexta* (Figure 7A, bioassay 3). Two weeks after challenging the plants with *Ma. sexta* (5 weeks after challenging the plants with *M. incognita*), we assessed the total number of eggs.

### Statistical analysis

All datasets were analyzed using the software *R* (version 3.1.2). To analyze gene expression, jasmonate levels, and metabolite accumulation, we used two-way analysis of variance (ANOVA) with treatment (T), time point (t), and their interaction as fixed factors. Following two-way ANOVAs, one-way ANOVAs with treatment as fixed factor were performed at each of the specific time points (3, 7, and 21 d). For *Ma. sexta* larval weight data, we used two-way ANOVAs with treatment (T), feeding period (F), and their interaction as fixed factors. For the relative abundance of *M. incognita* DNA dataset, we used two-way ANOVAs with treatment (T), time point (t), and their interaction as fixed factors. Normality and homogeneity of variance were verified using Shapiro–Wilk and Levene’s tests, respectively. When data did not meet any of the assumptions of ANOVA, square-root transformations were applied. Tukey’s test was used for overall comparisons among treatment groups within time points. Student’s *t* test was used for pairwise comparisons. OriginPro (version 2020b) was used for graphing.

### Data statement

The metabolomics datasets produced in this study have been deposited in the MetaboLights database, under the identification code MTBLS2507: www.ebi.ac.uk/metabolights/MTBLS2507

### Accession numbers

The accession numbers of the genes analyzed are displayed in Supplemental Table S4.

## Supplemental data

The following materials are available in the online version of this article.

**Supplemental Figure S1.** Expression levels of JA-responsive marker genes.

**Supplemental Figure S2.** Mass spectra and structures of the predicted metabolites.

**Supplemental Figure S3.** Relative intensity of *m*/*z* features selected at 3 d, without a predicted identity.

**Supplemental Figure S4.** Relative intensity of *m*/*z* features selected at 7 d, without a predicted identity.

**Supplemental Figure S5.** The levels of JA and JA-Ile in roots of grafts.

**Supplemental Table S1.** ANOVA table corresponding to data in Figures 1B, and 7B.

**Supplemental Table S2.** ANOVA table corresponding to data in Figures 2, 3, and 5.

**Supplemental Table S3.** The *m/z* features with the largest contribution to the total variance in the PCA.

**Supplemental Table S4.** Primer sequences used for the RT-qPCR analysis.

**Supplemental Material and Methods S1.** Metabolites extraction and analysis.

**Supplemental Material and Methods S2**. Data processing of the liquid chromatography mass spectrometry.

## Supplementary Material

kiab368_Supplementary_Data

## Notes

A.M.M. and N.M.vD. planned and designed the research. A.M.M. and C.M.M. performed experiments. A.M.M., C.M.M., A.M., A.W. and F.V. analyzed data. A.M.M. and N.M.vD. wrote the manuscript with input from all the authors.

The author responsible for distribution of materials integral to the findings presented in this article in accordance with the policy described in the Instructions for Authors (https://academic.oup.com/plphys/pages/general-instructions) is Ainhoa Martínez-Medina (ainhoa.martinez@irnasa.csic.es).
