# Supplementary material for: Leaf herbivory counteracts nematode-triggered repression of jasmonate-related defenses in tomato roots
Source: Plant Physiol. 2021 Aug 26;187(3):1762–78. doi: 10.1093/plphys/kiab368 (PMC8566281; doi:10.1093/plphys/kiab368)
Supplement: kiab368_Supplementary_Data [file kiab368_supplementary_data.pdf]

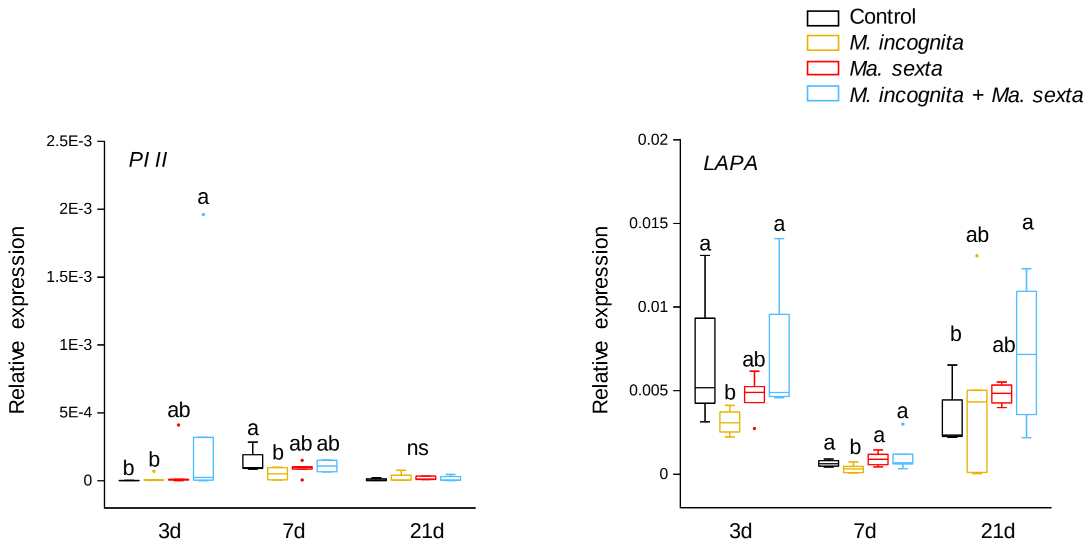

Supplemental Figure S1: Expression levels of JA-responsive marker genes. The expression levels of the JA-responsive marker genes *PI II* (*PROTEINASE INHIBITOR II*) and *LAPA (LEUCINE AMINOPEPTIDASE A)* were analysed in roots of plants that were challenged with *M. incognita* or *Ma. sexta* alone or in combination, and in unchallenged control plants. Gene expression were analysed 3, 7 and 21 days after *M. incognita* inoculation. Results are normalized to the *SlEF* gene expression levels. Box plots represent the interquartile range (IQR), the bisecting line represents the median, the whiskers represent 1.5 times the IQR, the dots represent outlier points, and the data are from 5 individual plants. At each specific time point, different letters indicate differences between treatments (ANOVA,Tukey's test $P < 0.05$). ns: not significant.

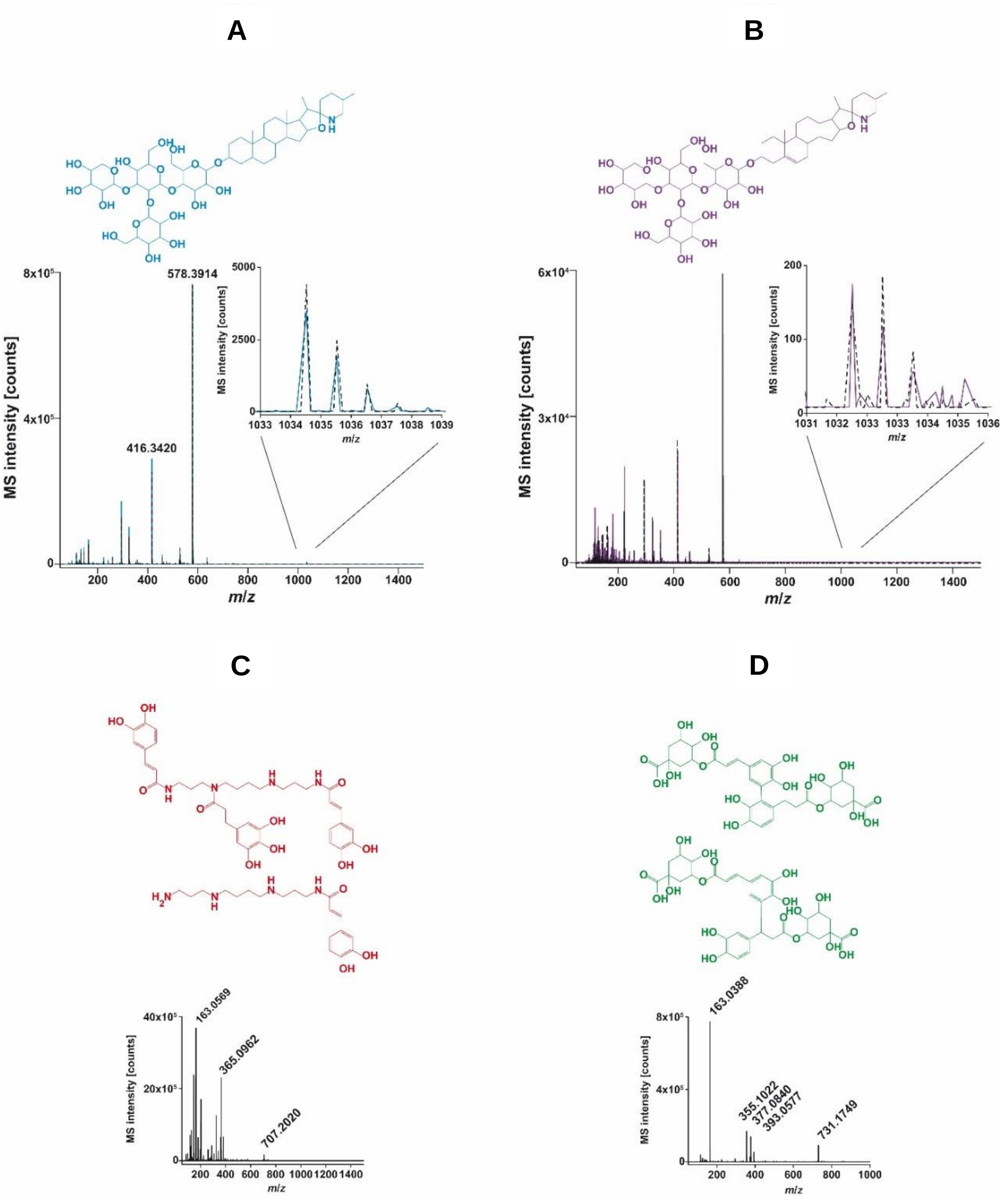

Supplemental Figure S2. Mass spectra and structures of the predicted metabolites. (A) α-tomatine; (B) α-dehydrotomatine; (C) a phenylpropanoid-polyamine conjugate; and (D) a chlorogenic acid dimer. In (A) and (B) the spectra of the compounds present in the root extracts are shown in color; spectra of the commercial α-tomatine (Extrasynthese, Lyon, France) which contains α-dehydrotomatine as impurity are shown in black.

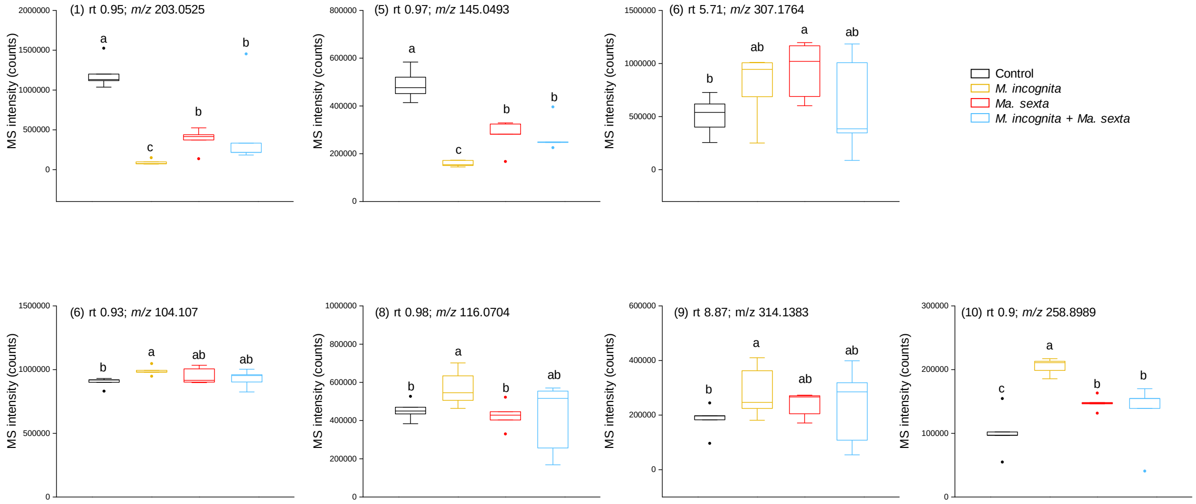

Supplemental Figure S3. Relative intensity of *m*/*z* features selected at 3 days, without a predicted identity. Metabolites were analysed in roots of tomato plants that were challenged with *M. incognita* or *Ma. sexta* alone or in combination, and in not challenged control plants at 3 days after *M. incognita* inoculation. Box plots represent the interquartile range (IQR), the bisecting line represents the median, the whiskers represent 1.5 times the IQR, the dots represent outlier points, and the data are from 5 individual plants. Different letters indicate differences among treatments (ANOVA, Tukey's test; *P*< 0.05). ns: not significant. Features are identified by the retention time (rt, in minutes) and mass to charge ratio (*m/z*). The number between brackets in every feature corresponds with the numbers in Figure 4A, and Supplementary Table S2.

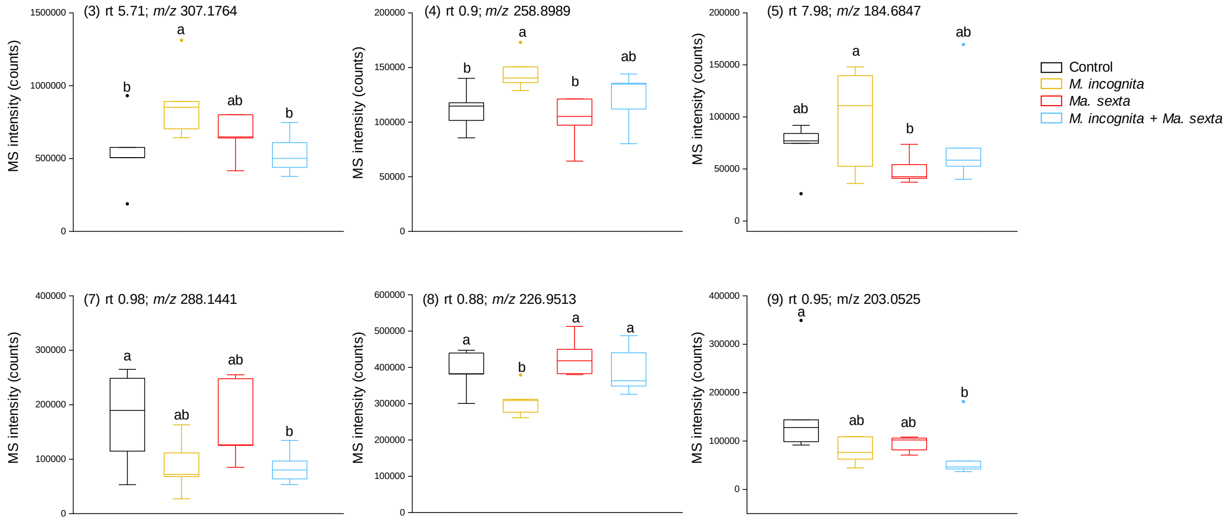

Supplemental Figure S4. Relative intensity of *m/z* features selected at 7 days, without a predicted identity. Metabolites were analysed in roots of tomato plants that were challenged with *M. incognita* or *Ma. sexta* alone or in combination, and in not challenged control plants at 7 days after *M. incognita* inoculation. Box plots represent the interquartile range (IQR), the bisecting line represents the median, the whiskers represent 1.5 times the IQR, the dots represent outlier points, and the data are from 5 individual plants. Different letters indicate differences among treatments (ANOVA,Tukey's test; $P < 0.05$). ns: not significant. Features are identified by the retention time (rt, in minutes) and mass to charge ratio (*m/z*). The number between brackets in every feature corresponds with the numbers in Figure 4B, and Supplementary Table S2.

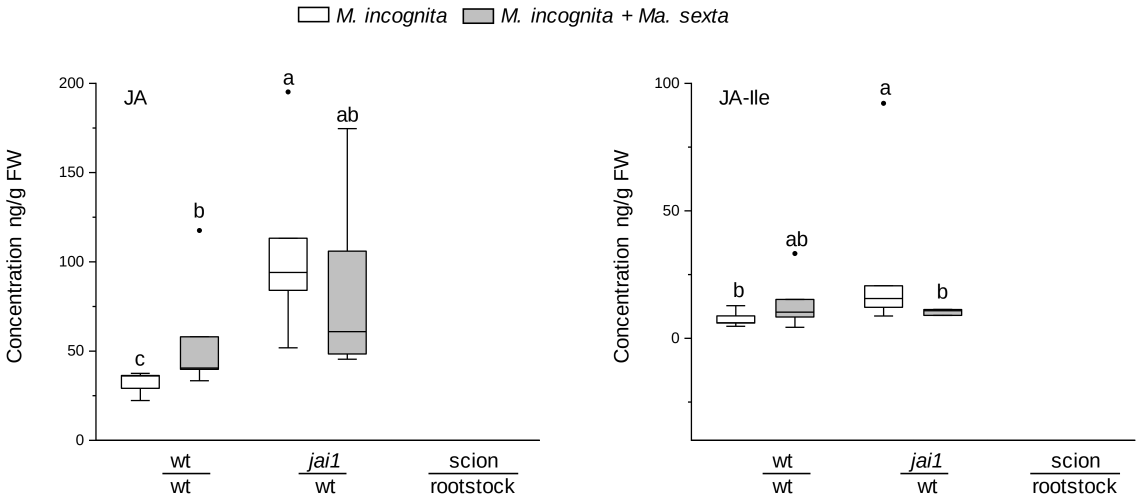

Supplemental Figure S5. The levels of JA and JA-Ile in roots of grafts. Grafts were made with rootstocks of the wild type (wt) Castlemart and scions of the wild type Castlemart (wt/wt) or the jasmonate perception compromised line *jai1 (jai*/wt). Grafts were challenged with *M. incognita* alone (*M. incognita*) and in roots of plants that were also shoot-challenged with *Ma. sexta* (*M. incognita* + *Ma. sexta*). The levels of JA and JA-Ile were analysed 21 days after *M. incognita* inoculation. Box plots represent the interquartile range (IQR), the bisecting line represents the median, the whiskers represent 1.5 times the IQR, the dots represent outlier points, and the data are from 5 individual plants. Different letters indicate differences between treatments (ANOVA, Tukey's test; $P < 0.05$).

Supplemental Table S1: ANOVA table corresponding to data in Figures 1B and 7B. Result of two-way ANOVA for the weight of *Manduca sexta* larvae feeding from plants that were also challenged or not with *Meloidogyne incognita*, with treatment (T), feeding period (F), and their interaction as model explanatory factors; and the relative abundance of *M. incognita* DNA in plants that were also challenged or not with *Ma. sexta*, with treatment (T), time point (t), and their interaction as model explanatory factors. Statistically significant effects are indicated in bold. The sample size for larval weight was 10, and for the relative abundance of *M. incognita* was 5.

	Treatment (T)	*Feeding Period (F)*	*T*F*
Variable	*P value*	*P value*	*P value*
Larval weight	0.225	0.484	0.078
	Treatment (T)	*Time point (t)*	*T*t*
Variable	*P value*	*P value*	*P value*
Relative abundance of *M. incognita* DNA	**0.047**	**0.05**	**0.042**

Supplemental Table S2: ANOVA table corresponding to data in Figures 2, 3 and 5. Results of two-way ANOVAs for gene expression, jasmonate levels and metabolite accumulation upon *Manduca sexta* and *Meloidogyne incognita* infection, with treatment (T), time point (t), and their interaction as model explanatory factors. Statistically significant effects are indicated in bold. The sample size for variables was 5.

	Treatment (T)	Time point (t)	T*t
Variable	*P value*	*P value*	*P value*
LoxD	**0.008**	**<0.001**	0.067
AOS1	**<0.001**	**<0.001**	**<0.001**
AOS2	**<0.001**	**<0.001**	0.538
AOC	**<0.001**	**0.001**	0.295
OPR3	**<0.001**	**<0.001**	0.355
LOXA	**0.002**	0.076	0.313
AOS3	**0.026**	0.155	0.288
DES	**0.048**	**<0.001**	**<0.001**
OPDA	**0.012**	**0.045**	0.623
JA	**0.049**	**<0.001**	**0.039**
JA-Ile	0.441	**<0.001**	0.489
α-tomatine	0.731	0.176	0.814
α-dehydrotomatine	0.206	0.492	0.180
Phenylpropanoid-polyamine conjugate	**0.05**	**0.015**	0.151
Chlorogenic acid dimer	**0.049**	0.723	0.161

Supplemental Table S3: The *m/z* features with the largest contribution to the total variance in the PCA. We set an arbitrary threshold of ±0.1 for the loading values in PC1 at 3 days and in PC2 at 7days (see Fig. 4 of the main document). All loadings with values > 0.1 and <-0.1 are shown. Colours of the different loadings correspond to the colours of the predicted metabolites in the loading plots displayed in figure 4 of the main document. Loadings without a predicted molecule are shown in black. Mass spectra and structures of the predicted molecules are shown in Supplemental Figure S2. Features ID sharing the same super-index at 3 and 7 days were found at both time points

3 days

Feature ID	Loading value on PC1	Average retention time [min]	Average *m/z* value	Predicted compound
1[¥]	-0.2989	0.95	203.0525	
2	-0.19448	0.96	365.1057	Phenylpropanoid_polyamine_conjugate
3	-0.19044	5.25	163.0387	Chlorogenic_acid_dimer
4	-0.17292	0.97	163.06	Phenylpropanoid_polyamine_conjugate
5	-0.13912	0.97	145.0493	
6[β]	0.169957	5.71	307.1764	
7	0.134018	0.93	104.107	
8	0.129053	0.98	116.0704	
9	0.11559	8.87	314.1383	
10	0.109279	0.9	258.8989	

Feature ID	Loading value on PC2	Average retention time [min] 0.97	Average *m/z* value	Predicted compound
1	-0.28216	8.65	578.4056	α-tomatine
2	-0.1695	8.41	576.3901	α-dehydrotomatine
3[β]	-0.13989	5.71	307.1764	
4	-0.12466	0.9	258.8989	
5	-0.11157	7.98	184.6847	
6	0.183936	0.96	365.1057	Phenylpropanoid_polyamine_conjugate
7	0.182069	0.98	288.1441	
8	0.144798	0.88	226.9513	
9[¥]	0.143933	0.95	203.0525	
10	0.117518	0.97	163.06	Phenylpropanoid_polyamine_conjugate

Supplemental Table S4: Primer sequences used for the qPCR and RT-qPCR analysis

ID	Target Gene	Primer (5´-3´)
U37840	*LIPOXYGENASE D* (*LOXD*)[1]	GACTGGTCCAAGTTCACGATCC<br>ATGTGCTGCCAATATAAATGGTTCC
AJ271093	*ALLENE OXIDE SYNTHASE 1* (*AOS1*)[1]	CACCTGTTAAACAAGCGAAAC<br>GACCTGGTGGCATGTTCGT
AF230371	*ALLENE OXIDE SYNTHASE 2* (*AOS2*)[1]	AGATTTTCTTCCCGAATATGCTGAA<br>ATACTACTGATTTCATCAACGGCAT
AF384374	*ALLENE OXIDE CYCLASE* (*AOC*)[1]	GCACGAAGAAGAGAAGAAAGGAGAT<br>CGGTGACGGCTAGGTAAGTTTC
AJ278332	*12-OXOPHYTODIENOIC ACID REDUCTASE 3* (*OPR3*)[1]	TTGGCTTAGCAGTTGTTGAAAG<br>TACGTATCGTGGCTGTGTTACA
U09026	*LIPOXYGENASE A* (*LOXA*)[1]	GGTTACCTCCCAAATCGTCC<br>TGTTTGTAACTGCGCTGTG
AF454634	*ALLENE OXIDE SYNTHASE 3* (*AOS3*)[1]	GCGGAGGAGTTCAATCCAG<br>CGCATGAAAAACTCCACAACC
AF317515	*DIVINYL ETHER SYNTHASE* (*DES*)[1]	CCGGATGAGTTTGTACCTGA<br>ATCTTTGCCTGGACATTGCT
X14449	*ELONGATION FACTOR 1A* (*SlEF*)[1]	GATTGGTGGTATTGGAACTGTC<br>AGCTTCGTGGTGCATCTC
MINC06773a	*M. incognita ACTIN*[2]	GATGGCTACAGCTGCTTCGT<br>GGACAGTGTTGGCGTAAAGG
K03291	*PROTEINASE INASE INHIBITOR II (PI II)*[3]	GAAAATCGTTAATTTATCCCAC<br>ACATACAAACTTTCCATCTTTA
U50151	*LEUCINE AMINOPEPTIDASE A (LAPA)*	ATCTCAGGTTTCCTGGTGGAAGGA<br>AGTTGCTATGGCAGAGGCAGAG

[1]López-Ráez et al. (2010); [2]Teillet et al. (2013); [3]Martinez-Medina et al. (2013); [4]Fowler et al. (2009).

Supplemental Material and Methods S1: Metabolites extraction and analysis

We extracted 100 mg fresh ground root tissue of each sample in 1 ml of extraction buffer (75% methanol acetate buffer; pH 4.8; diluted 1:5, v : v). The samples were homogenized for 5 min at 30 Hz using a ball mill (Retsch mixer mill MM 400), and subsequently centrifuged (14.000 rpm, 10 min, 4°C). The supernatant was collected in a 2 ml Eppendorf tube. We repeated the extraction procedure with the remaining pellet and combined the supernatant with the first one. We centrifuged (14.000 rpm, 5 min, 4°C) all extracts, transferred 200 µl of each to an HPLC vial and added 800 µl extraction buffer, resulting in a 1:5 dilution. We performed chromatographic separation of all diluted extracts by injecting 2 µl on a Thermo Scientific Dionex UltiMate 3000 UPLC (Thermo Scientific Dionex, Sunnyvale, USA), equipped with a C18 column (Acclaim RSLC 120 C18, 2.2 µm, 120 Å, 2.1 x 150 mm, Thermo Fisher Scientific). We applied the following binary elution gradient at a column temperature of 40°C; and a flow rate of 0.4 ml min^{-1}: A: water/formic acid (0.05% v/v), solvent B: acetonitrile/formic acid (0.05% v/v); gradient for solvent B: 0 min 5%, 2 min 5%, 7 min 30%, 12 min 35%, 15 min 95%, 17 min 95%, 19 min 5%, 23 min 5%. Metabolites were analysed on a quadrupole/time-of-flight mass spectrometer (qToF-MS; Bruker maXis impact HD; Bruker Daltonik, Bremen, Germany) with an electrospray ionization source operated in positive mode. Instrument settings were as follows: capillary voltage, 2,500 V; nebulizer, 2.5 bar; dry gas temperature, 220°C; dry gas flow, 11 L min^{-1}; scan range, 50–1000 *m*/*z*; acquisition rate, 1 Hz. We used sodium formate clusters (10 mM solution of NaOH in 50/50% [v/v] isopropanol/water containing 0.2% formic acid) to perform mass calibration. A quality control sample (mix of root extracts) and a commercial standard of α-tomatine (Extrasynthese) were injected with the same conditions described above but the scan range was modified to 50–1500 *m*/*z*.

Supplemental Material and Methods S2: Data processing of the liquid chromatography mass spectrometry

We transformed the LC-qToF-MS raw data into *.mzXML* files with the programs CompassXport and DataAnalysis v4.2 SR2 (Bruker Daltonik GmbH). We converted the *.mzXML* files into *.abf* files with the software Reifycs Abf Converter (RIKEN, https://www.reifycs.com/AbfConverter/). We deconvoluted the LC-qToF-MS data stored in the *.abf* files with the software MS-DIAL v3.08 from RIKEN (Lai 2018). Processing parameters were as follows: soft ionization, centroid data type, positive ion mode, (*i*) data collection: mass accuracy MS1 = 0.01 Da, retention time 0.7–14 min, mass range 45-1005 Da; (*ii*) peak detection: minimum peak height (amplitude) = 1,000, mass slice width = 0.1 Da, smoothing method: linear weighted moving average, smoothing level = 3, minimum peak width = 5; (*iii*) alignment: retention time tolerance = 0.05 min, MS1 tolerance = 0.015 Da. We normalized the data using the total ion chromatogram function of MS-DIAL. We exported the alignment matrix as *.csv* file. The numbers in this matrix represent features defined by an average retention time and an average *m*/*z* value. We used the matrix for computing multivariate statistical analyses with the software *R* (v x64 3.5.1, *R* Foundation for Statistical Computing). We worked with the package *muma* for metabolomics (http://www.eurekaselect.com/107837) to calculate principal component analysis (PCA). We applied the function *pareto* for scaling the data.
